# Metagenomic next-generation sequencing: A promising tool for diagnosis and treatment of suspected pneumonia in rheumatic patients with acute respiratory failure: Retrospective cohort study

**DOI:** 10.3389/fcimb.2022.941930

**Published:** 2022-08-03

**Authors:** Yan Shi, Jin-Min Peng, Han-Yu Qin, Bin Du

**Affiliations:** Department of medical ICU, Peking Union Medical College Hospital, Peking Union Medical College and Chinese Academy of Medical Sciences, Beijing, China

**Keywords:** metagenomic next-generation sequencing (mNGS), pneumonia, rheumatic patients, acute respiratory failure, bronchoalveolar lavage fluid (BALF), clinical decision-making

## Abstract

**Background:**

The effectiveness of metagenomic next-generation sequencing (mNGS) in respiratory pathogen detection and clinical decision-making in critically rheumatic patients remains largely unexplored.

**Methods:**

A single-center retrospective study of 58 rheumatic patients who were admitted to ICU due to suspected pneumonia with acute respiratory failure if they underwent both bronchoalveolar lavage fluid specimen mNGS and combined microbiological tests (CMTs) was conducted to compare their diagnostic performance, using clinical composite diagnosis as the gold standard. Treatment modifications based on mNGS results were also reviewed.

**Results:**

Forty-three patients were diagnosed with microbiologically confirmed pneumonia and 15 were considered as a non-infectious disease. mNGS outperformed CMTs in the accurate diagnosis of infectious and non-infectious lung infiltration (98.1% [57/58] vs. 87.9% [51/58], *P* = 0.031). A total of 94 causative pathogens were defined by the gold standard and 27 patients had polymicrobial pneumonia. The sensitivity of pathogen detection and complete concordance with the gold standard by mNGS exceeded those by CMTs (92.6% [87/94] vs. 76.6% [72/94], *P* < 0.001 and 72.1% [31/43] vs. 51.2% [22/43], *P* = 0.004, respectively). Moreover, 22 pathogens were detected only by mNGS and confirmed by orthogonal test. Accordingly, the etiological diagnosis changed in 19 cases, and the empirical treatment improved in 14 cases, including 8 cases of rescue treatment and 11 of antibiotics de-escalation. At the pathogen-type level, both methods were comparable for bacteria, but mNGS was advantageous to identify viruses (accuracy: 100% vs. 81%, *P* = 0.004). For *Pneumocystis jirovecii* detection, mNGS improved the sensitivity compared with Gomori’s methenamine silver stain (91.7% vs. 4.2%, *P* < 0.001) and was higher than polymerase chain reaction (79.2%), but the difference was not significant (*P* = 0.289). In terms of *Aspergillus*, the better sensitivity with a combination of culture and galactomannan test than that with mNGS was found (100% vs. 66.7%, *P* = 0.033).

**Conclusions:**

mNGS has an excellent accuracy in etiological diagnosis and pathogen detection of suspected pneumonia in critically rheumatic patients, which has potential significance for clinical decision-making. Its superiority to different types of pathogens depends on the comprehensiveness of CMTs.

## Introduction

Pneumonia remains the major cause of morbidity and mortality in rheumatic patients. The majority of patients present with acute respiratory failure (ARF) and require intensive care unit (ICU) admission, with mortality up to 70% (Janssen et al., 2002; Falagas et al., 2007). This population is vulnerable to a wide range of pathogens and superimposed infections, so early and correct assessment of microbial etiology is very important for appropriate antimicrobial therapy and favorable prognosis of severe infections (Falagas et al., 2007; Feng et al., 2010; Azoulay et al., 2020). However, the pathogen diversity and low microbial loads in immunocompromised patients (ICPs) pose challenges to traditional microbiological methods due to their limitations in the sensitivity, speed, and breadth of pathogen detection (Chiu and Miller, 2019; Azoulay et al., 2020). Moreover, compared with other ICPs, the etiology of rheumatic patients with lung infiltrations is more complex. The initial clinical manifestation of pneumonia in this population may be similar to those of non-infectious lung infiltrates (e.g., rheumatic disease activity related lung involvement or drug-related toxicity), which also greatly increases the difficulty of diagnosis (Janssen et al., 2002; Falagas et al., 2007; Papiris et al., 2016). Given the serious clinical consequences of mistakenly treating an infection with immunosuppression or a flare with antibiotics, more sensitive techniques are needed for precise and timely diagnosis and treatment (Feng et al., 2010; Azoulay et al., 2020).

Metagenomic next-generation sequencing (mNGS) is a nucleic acid sequencing technique with high-throughput capacity. Unlike polymerase chain reaction (PCR)–based and 16*S* ribosomal RNA gene–based sequencing approaches, it is an unbiased method independent of etiological hypothesis and theoretically permits the identification of all pathogens in a single assay. Due to fast turnaround time and high sensitivity, mNGS could emerge as a promising tool, especially for pathogen detection of rare, novel, and complicated infectious diseases (Chiu et al., 2019; Gu et al., 2019). In recent years, its diagnostic performance has been studied in different patient populations with different types of infections (Prachayangprecha et al., 2014; Gu et al., 2019; Wilson et al., 2019; Zhang et al., 2020). However, only a few studies have described its utility in detecting respiratory pathogens in ICPs (Parize et al., 2017; Langelier et al., 2018; Pan et al., 2019; Zhang et al., 2019), and most of them have only focused on bacteria or viruses (Parize et al., 2017; Langelier et al., 2018). For instance, in a study of 101 heterogeneous ICPs, where pneumonia represented only less than 30%, Parize et al. demonstrated that clinically relevant viruses and bacteria were detected in a significantly higher proportion of patients with NGS than conventional methods (36% vs. 11%, *P* < 0.001) (Parize et al., 2017). Likewise, a study of 22 human stem cell transplant recipients reported an improved capacity of mNGS to detect viruses and bacteria as pneumonia pathogens (Langelier et al., 2018).

It was worth noting that the pathogen profiles were diverse in different ICPs populations, which might affect its diagnostic performance (Reynolds et al., 2010; Bitar et al., 2014; Di Franco et al., 2017). For example, the prominent feature of patients with hematologic malignancies was the depressed neutrophil function, which leads to a peculiar vulnerability to bacteria and fungi (Pagano et al., 2012; Pergam, 2017). In addition, the pathogen profiles of transplant recipients were diverse in different periods after transplantation. Meanwhile, for rheumatic patients, a variety of immune disorders related to its internal diseases and the frequent use of immunosuppressive drugs, together with the rheumatic diseases related to lung involvement, make them more vulnerable to various opportunistic pathogens and superimposed infections (Falagas et al., 2007; Papiris et al., 2016; Di Franco et al., 2017). Therefore, in clinical practice, it is more meaningful to evaluate the effectiveness of different technologies for homogeneous populations, especially for rheumatic patients who are more prone to varied etiology of ARF and complicated infections. Moreover, the purpose of ordering mNGS was to provide additional information for clinical decision-making, which has rarely been reported in previous studies (Miao et al., 2018; Li et al., 2020) and demands further investigation.

The purpose of this study was to evaluate the efficacy of bronchoalveolar lavage fluid (BALF) specimen mNGS on etiological diagnosis and pathogen identification in rheumatic patients with suspected pneumonia, by comparing the results of mNGS, combined microbiological tests (CMTs), and clinical composite diagnosis standard (i.e., the gold standard). We also reviewed treatment modifications based on mNGS results to explore its implications on clinical decision-making.

## Methods

### Study design and patients

All adult rheumatic patients who were admitted to the medical ICU of Peking Union Medical College Hospital (Beijing, China) from January 2019 to December 2020 due to suspected pneumonia with ARF (defined as PaO_2_/FiO_2_ ratio of < 300 mmHg) were retrospectively reviewed if they underwent BALF mNGS within 48 h after ICU admission. The exclusion criterion was that mNGS and CMTs were not paired, that is, they were not performed simultaneously or on the same day.

This study was approved by the Research Ethics Committee of our hospital. Individual consent for this retrospective analysis was waived.

### Data collection

Demographic and clinical data were extracted from electronic medical records (EMRs), including age, gender, type of rheumatic disease, steroids or immunosuppressants used, severity of illness by the Acute Physiology and Chronic Health Evaluation (APACHE) II and Sequential Organ Failure Assessment (SOFA) score on ICU admission, empirical antibiotics, results of CMTs and mNGS, treatment modifications, life-sustaining therapies, and outcome.

### Combined microbiological tests and interpretation of results

All patients uniformly underwent CMTs, including culture for bacteria, fungi, and *Mycobacterium*; special stain for *Mycobacterium*, *Cryptococcus*, and *P. jirovecii*; serological antibody for atypical respiratory pathogens; antigen detection for fungi, Influenza A/B and *Legionella pneumophila*; direct examination for mold and PCR test for atypical respiratory pathogens, *Mycobacterium*, *P. jirovecii*, and viruses, but specific PCR items for virus detection were performed according to the clinician’s discretion, which mainly carried out in the flu season or when viral pneumonia was highly suspected, including influenza virus, parainfluenza virus, rhinovirus, adenovirus, and respiratory syncytial virus (RSV).

The interpretations of microbiological results and definitions of pneumonia caused by a specific pathogen were as described in the literature (Xue et al., 2016; Lee et al., 2017; Donnelly et al., 2020) (**Supplementary Table S1**). Lung biopsy has been considered as the gold standard for the diagnosis of fungal and cytomegalovirus (CMV) pneumonia, whereas it is defective in its invasiveness and is hard to use in clinical experiences. Therefore, this study mainly adopted the clinical diagnostic criteria. The diagnosis of fungal pneumonia referred to the criteria for probable invasive fungal disease by the European Organization for Research and Treatment of Cancer and the Mycoses Study Group (EORTC/MSG) (Donnelly et al.,2020). Similarly, considering that CMV not only can induce the exacerbation of rheumatic disease but also may present as severe viral pneumonia, CMV infection may be aggressively diagnosed in ICPs with very high or increasing CMV DNA levels based on CMV DNA quantitation in BALF, in addition to clinical and radiographic evidence (Cunha et al., 2010; Xue et al., 2016; Lee et al., 2017).

### Procedures of BALF mNGS and criteria for a positive result

BALF samples were immediately sent to BGI clinical laboratory Co., Ltd. for nucleic acid extraction, library construction, high-throughput sequencing, and bioinformatics analysis. RNA sequencing was performed in the flu season or when clinicians highly suspected viral pneumonia. The turnaround time was about one working day. Detailed procedures were given in Appendix 1.

In view of the lack of standard methods to interpret mNGS results and the diversity of reporting parameters between different sequencing platforms, in our practice, we adopted standards derived and revised from the previous literature on the BGISEQ platform to define clinically relevant microorganisms (CRMs) (Miao et al., 2018; Fang et al., 2020; Li et al., 2020). For bacteria (mycobacteria excluded), fungi (molds excluded), viruses, and parasites, a microbe was considered as CRM when its relative abundance at the species level was >30%, and there existed literature evidence of pathogenicity in the lungs. *Mycobacterium* detected by mNGS was considered as CRM when the stringently mapped read number (SMRN) at the species level was >3. Molds with literature-proven pulmonary pathogenicity were considered as CRMs when the SMRN at the species level was >10. Microorganisms that cannot cause pneumonia, including *Corynebacterium*, coagulase-negative *Staphylococci* and *Neisseria*, which are normally parasitic in the human oropharynx, were not considered CRMs regardless of their relative abundance. The sequencing data of CRMs were listed in **Supplementary Table S2**.

Microbes solely identified by mNGS were considered as new potential pathogens if they showed literature evidence of pathogenicity and were consistent with clinical presentation (Langelier et al., 2018; Chen et al., 2020; Fang et al., 2020). These were based on strict clinical criteria and combined with multiple-clinician adjudication. In addition, orthogonal confirmation of positive tests for *P. jirovecii*, atypical pathogens, and viruses on mNGS has been performed with PCR. Meanwhile, repeated mNGS assay was performed on microbes identified by CMTs alone, but the results were not used to evaluate the diagnostic performance of mNGS.

### Gold standard for causative pathogens

The final determination of causative pathogens was based on the clinical composite diagnostic criteria (i.e., the gold standard), which was established by the comprehensive analysis of CMTs and mNGS results and other relevant information (such as clinical suspicion diagnosis, clinical manifestation, laboratory tests, imaging findings, and treatment effect observations) (Miao et al., 2018; Chen et al., 2020; Fang et al., 2020; Li et al., 2020). Meanwhile, microorganisms detected only by CMTs or mNGS but not regarded as causative pathogens by the gold standard were defined as false positive. Two intensivists with expertise in the management of infection in ICPs (YS and JMP) independently reviewed the EMRs of each patient, and any disagreement was resolved through in-depth discussion.

### Statistics

Following the extracted data, 2 × 2 contingency tables were derived to determine the sensitivity, specificity, positive predictive value (PPV), negative predictive value (NPV), and accuracy. All statistics were reported as absolute values with their 95% confidence interval (CI) and determined using the Wilson’s method. Given that mixed infections were common in ICPs, we also evaluated the diagnostic performance of different methods by the complete concordance with the gold standard. That is, only when all pathogens were detected can it be considered as a correct diagnosis. The McNemar test was used to compare the pathogen detection rate of each diagnostic procedure. Data analysis was performed with SPSS 22.0. P < 0.05 was considered statistically significant.

## Results

### Patient characteristics

During the study period, 103 rheumatic patients were admitted to ICU due to suspected pneumonia with ARF. Among them, 62 patients who underwent BALF mNGS within 48 h after ICU admission were eligible. Except for four patients who were not paired of mNGS and CMTs, 58 (mean age, 51.3 years, 65.5% females) were included in the final analysis. A total of 13 patients underwent viral PCR and RNA sequencing.

The clinical characteristics were summarized in **Table 1**. Systemic lupus erythematosus (SLE), idiopathic inflammatory myopathies, and systemic vasculitis were the most commonly rheumatic diseases (72% of patients). On ICU admission, the APACHE II and SOFA scores were 18.8 ± 5.3 and 7.1 ± 2.6, respectively. The median PaO_2_/FiO_2_ ratio was 167 mmHg and 51 (87.9%) patients received invasive mechanical ventilation. Fifty-two (89.7%) and 56 (96.6%) patients were exposed to antibiotics prior to ICU admission and BALF sample collection, respectively. Forty-one (70.7%) patients were prescribed three or more antibiotics and the most commonly used antibiotics were ceftriaxone, moxifloxacin, and ganciclovir combined with trimethoprim-sulfamethoxazole. Thirty-four patients (58.6%) died in the ICU.

**Table 1:** Characteristics of 58 critically rheumatic patients with suspected pneumonia.

Characteristic	Value
Age, years, mean (SD)	51.3 (17.1)
Sex-female, n (%)	38 (65.5)
Type of rheumatic disease, n (%)	
Systemic lupus erythematosus	19 (32.8)
Systemic vasculitis	11 (19.0)
Idiopathic inflammatory myopathies	12 (20.7)
Rheumatoid arthritis	5 (8.6)
Others^†^	11 (19.0)
Specific therapy for rheumatic disease, n (%)	
Systemic corticosteroids	58 (100)
Cytotoxic or immunosuppressants	19 (32.8)
Disease severity at admission	
APACHE II score, mean (SD)	18.8 (5.3)
SOFA score, mean (SD)	7.1 (2.6)
PaO_2_/FiO_2_ ratio, mmHg, median (IQR)	167 (103, 215)
HFNC or noninvasive ventilation, n (%)	7 (12.1)
Invasive mechanical ventilation, n (%)	51 (87.9)
Vasopressor, n (%)	28 (48.3)
Laboratory findings	
White blood cell, 10^9^/L, median (IQR)	7.1 (3.2, 12.0)
Lymphocyte, 10^6^/L, median (IQR)	470 (235,770)
CD4^+^ T cell, 10^6^/L, median (IQR)	99 (55, 204)
Outcomes	
ICU death, n (%)	34 (58.6)
ICU LOS, days, median (IQR)	12.5 (6.3, 23.8)
Hospital death, n (%)	35 (60.3)
Hospital LOS, days, median (IQR)	22 (9, 31)

APACHE, Acute Physiology and Chronic Health Evaluation; HFNC, high-flow nasal cannula; ICU, intensive care unit; IQR, interquartile range; LOS, length of stay; SD, standard deviation; SOFA, Sequential Organ Failure Assessment.

^†^Including adult-onset Still’s disease (n = 3); interstitial pneumonia with autoimmune features (n = 3); Sjögren’s syndrome (n = 2); mixed connective tissue disease (n = 1); systemic sclerosis (n = 1); undifferentiated connective tissue disease (n = 1).

### Diagnostic performance of CMTs and mNGS

#### Comparisons for differentiating pneumonia from non-infectious etiologies

According to the gold standard, 43 patients were diagnosed with microbiologically confirmed pneumonia (hospital-acquired [*n* = 19], community-acquired [*n* = 24]) and 15 patients were diagnosed with a non-infectious disease, including five with dermatomyositis-related interstitial lung disease (ILD), nine with SLE activity-related lung involvement (2 of ILD, 5 of diffuse alveolar hemorrhage and 2 of acute cardiogenic pulmonary edema), and one with systemic sclerosis-related ILD (**Supplementary Table S2**).

In terms of etiological diagnosis of ARF, mNGS results were positive in all 43 patients with pneumonia and were false positive in 1 of 15 without pneumonia (No. 23), corresponding to 100% (95% CI, 89.7%–100%) of sensitivity, 93.3% (95% CI, 66.0%–99.6%) of specificity, 97.7% (95% CI, 86.5%–99.8%) of PPV, 100% (95% CI, 73.2%–100%) of NPV, and 98.3% (95% CI, 88.7%–99.1%) of accuracy. On the contrary, CMTs failed to identify *P. jirovecii* in 5 of 43 cases with pneumonia (Nos. 18, 19, 26, 33, and 40), and 2 of 15 cases without pneumonia were false-positive (Nos. 23 and 49), corresponding to 88.4% (95% CI, 74.1%–95.6%) of sensitivity, 86.7% (95% CI, 58.4%–97.6%) of specificity, 95% (95% CI, 81.7%–99.1%) of PPV, 72.2% (95% CI, 46.4%–89.2%) of NPV, and 87.9% (95% CI, 71.3%–91.3%) of accuracy (**Supplementary Table S2**). As a result, mNGS outperformed CMTs in NPV and accuracy of etiological diagnosis (*P* = 0.016 and *P* = 0.031, respectively).

#### Comparisons for causative pathogen detection

A total of 94 causative pathogens were defined by the gold standard, including 21 bacteria (18 common bacteria, 2 *Mycobacterium tuberculosis*, and 1 *Mycoplasma pneumoniae*), 42 fungi (24 P*. jirovecii*, 15 *Aspergillus* spp., and 3 other fungi), and 31 viruses (17 CMV, 4 influenza virus, 1 parainfluenza* *virus, 1 RSV, and 8 others) (**Table 2**). Eighty-seven of the 94 causative pathogens were detected by mNGS, whereas 72 were detected by CMTs. In addition, 12 microbes were considered as false positive. Of which, five strains of *Acinetobacter baumannii* detected by both methods were considered as colonization (Nos. 6, 2 0, 23, 30, and 38), three strains of bacteria identified by mNGS alone were also considered as colonization (Nos. 29, 38, and 47), and the other four detected by CMTs alone were considered as false positive and the possible reasons included colonization (*P. jirovecii* in No. 50), possible contamination (*Aspergillus* in No. 49), and likelihood of latent infection (CMV in Nos.55 and 58) (**Table 2** and **Supplementary Table S2**). mNGS increased the sensitivity and accuracy for causative pathogen detection when compared with CMTs (92.6%, 95% CI, 84.8%–96.7% vs. 76.6%, 95% CI, 66.5%–84.5% and 85.8%, 95% CI, 73.5%–94.6% vs. 70.8%, 95% CI, 60.1%–79.8%, all *P* < 0.001, respectively), while the false-positive rate was comparable (*P* = 0.973).

**Table 2:** Causative pathogen and divergent identifications by mNGS and CMTs in 43 patients with microbiologically confirmed pneumonia.

Causative pathogen	All (n = 43)	Single pathogen(n = 16)	Mixed pathogens(n = 27)	*P* value	Detected by both methods	Detected by CMTs alone	Detected by mNGS alone
**Bacteria**	17 (40)	4 (25)	13 (48)	0.328	15	0	2
*A. baumannii*	6 (14)	1 (6)	5 (19)	0.386	6	0	0
*P. aeruginosa*	6 (14)	2 (13)	4 (15)	1.000	6	0	0
*M. tuberculosis*	2 (5)	0 (0)	2 (7)	0.522	2	0	0
Other bacteria^†^	7 (16)	1 (6)	6 (22)	0.229	5	0	2
**Fungi**	33 (77)	10 (63)	23 (85)	0.137	20	6	7
*P. jirovecii*	24 (56)	9 (56)	15 (56)	1.000	17	2	5
*Aspergillus* spp.	15 (35)	1 (6)	14 (52)	0.003	10	5	0
*Cryptococcus*	1 (2)	0 (0)	1 (4)	1.000	1	0	0
*Rhizopus *spp.	2 (5)	0 (0)	2 (7)	0.522	0	0	2
**Viruses**	23 (54)	2 (13)	21 (78)	<0.001	13	0	10
Cytomegalovirus^$^	17 (40)	1 (6)	16 (59)	0.001	16	0	1
Influenza A/B	4 (9)	0 (0)	4 (15)	0.279	2	0	2
Parainfluenza	1 (2)	1 (6)	0 (0)	0.372	0	0	1
RSV	1 (2)	0 (0)	1(4)	1.000	0	0	1
Other viruses^‡^	8 (19)	0 (0)	8 (30)	0.036	0	0	8

Data are presented as numbers (%) or numbers.

CMTs, combined microbiological tests; mNGS, metagenomic next-generation sequencing; RSV, respiratory syncytial virus.

^†^Including K. pneumoniae (n = 2), E. faecium (n = 1), Salmonella (n = 1), M. Pneumoniae (n = 1), S. pneumoniae (n = 1), and H. influenzae (n = 1).

^‡^ Including human herpes virus type 1 (HHV-1) (n = 5), HHV-6 (n = 1), human coronavirus 229 E (n = 1), and human coronavirus OC 43 (n = 1).

^$^The median cytomegalovirus (CMV) load was 2.52 × 10^5^ (7.4 × 10^4^, 5.5 × 10^5^) copies/ml in 17 CMV pneumonia patients.

Among 43 patients with confirmed pneumonia, mixed infection was presented in 27 cases with two pathogens in 12 cases and more than two pathogens in 15 cases. Viruses and *Aspergillus* spp. were more likely to be associated with polymicrobial pneumonia (**Table 2**). Mixed fungal–viral (*n* = 9), bacterial–fungal (*n* = 6), and bacterial–fungal–viral infections (*n* = 6) were the most common patterns of mixed infection. The pathogens detected by mNGS were completely matched the gold standard in 31 cases and partially matched in 12 cases, including six of missing partial pathogens (Nos. 1, 5, 7, 9, 13, and 55, mainly *Aspergillus* spp.) and six of additional colonized bacteria (Nos. 6, 20, 29, 30, 38, and 47). In terms of CMTs, complete and partial matching of the gold standard was found in 22 and 14 cases, respectively. In addition, CMT results were paradoxical with the gold standard in two cases (Nos. 6 and 20) and no pathogen was identified in the remaining five cases with pneumonia (Nos. 18, 19, 26, 33, and 40) (**Supplementary Table S2**). As a result, the complete concordance rate between mNGS and the gold standard for pathogen identification was better than that of CMTs (72.1%, 95% CI, 56.1%–84.2% vs. 51.2%, 95% CI, 35.7%–66.5%, *P* = 0.004), especially for co-pathogens (77.8%, 95% CI, 60.7%–88.6% vs. 55.6%, 95% CI, 43.1%–68.9%, *P* = 0.015).

#### Case evaluation of discrepant results between CMTs and mNGS

Twenty-two causative pathogens from 17 patients were only detected by mNGS and confirmed by orthogonal confirmation (excluded bacteria), including 13 viruses (human herpes virus type 1 [HHV-1] in Nos. 13, 20, 31, 47, and 58; influenza virus in Nos. 8 and 34; human coronavirus in Nos. 12 and 35; CMV in No. 20; parainfluenza virus in No. 6; RSV in No. 34; and HHV-6 in No. 20), 5 P*. jirovecii* (Nos. 18, 19, 26, 33, and 40), 2 *Rhizopus *spp. (Nos. 11 and 47), and 2 bacteria (*S. pneumoniae* in No. 31 and *H. influenzae* in No. 41). Among them, parainfluenza virus in No. 6 and RSV in No. 34 were not detected by CMTs because physicians did not order relevant PCR tests. In addition, three of the four patients diagnosed with influenza virus were exposed to influenza treatment before sample collection (Nos. 4, 8, and 34). In comparison, seven causative pathogens were identified by CMTs alone, including four with a positive serum/BALF galactomannan (GM) test (Nos. 1, 5, 7, and 9), two with a positive PCR for *P. jirovecii* (Nos. 1 and 13), and one with a positive culture for *Aspergillus* (No. 55). Among these mNGS false-negative cases, a repeat mNGS assay showed that three cases (Nos. 1, 5, and 9) had small SMRN of *Aspergillus* spp. (range from 3 to 5) without meeting our positive criteria and the remaining four were completely unidentifiable by mNGS (**Table 2** and **Supplementary Table S2**).

Some viruses with uncertain pulmonary pathogenicity were detected by mNGS alone (such as parvovirus and torque teno virus), which no subsequent orthogonal confirmatory tests were performed due to a lack of appropriate identification techniques, the real clinical significance needs further study.

#### Comparison of mNGS and CMTs in different pathogen type

Both methods were comparable for bacteria detection, but the diagnostic accuracy of mNGS for viruses was higher than that of CMTs (100%, 95% CI, 87.9%–100% vs. 81.0%, 95% CI, 62.6%–87.7%, *P* = 0.004), which was mainly due to its higher sensitivity to viruses other than CMV (100%, 95% CI, 69.9%–100% vs. 16.7%, 95% CI, 3.0%–49.1%, *P* < 0.001). Notably, there were subtle differences in identifying specific fungal species depending on the diagnostic tests used. In terms of *Aspergillus* spp. detection, culture, GM test, and mNGS were positive in 8, 13, and 10 patients respectively, indicating that the diagnostic sensitivity of GM was better than culture (86.7%, 95% CI, 58.4%–97.7% vs. 53.3%, 95% CI, 27.4%–77.7%, *P* = 0.043) and slightly higher than mNGS (66.7%, 95% CI, 38.7%–87.0%), but the difference was not significant (*P* = 0.095). In addition, a better sensitivity with a combination of culture and GM test than that with mNGS was found (100%, 95% CI, 74.7%–100% vs. 66.7%, 95% CI, 38.7%–87.0%, *P* = 0.033). For *P. jirovecii* detection, the Gomori’s methenamine silver stain (GMS), PCR test, and mNGS were positive in 1, 19, and 22 patients, respectively. As a result, mNGS significantly increased the diagnostic sensitivity when compared with GMS (91.7%, 95% CI, 71.5%–98.5% vs. 4.2%, 95% CI, 0.2%–23.1%, *P* < 0.001) and was higher than PCR (79.2%, 95% CI, 57.3%–92.1%), although the difference was not significant (*P* = 0.289) (**Supplementary Table S3**).

#### Potential implications of mNGS on diagnosis and treatment

Based on mNGS results, the initial diagnosis of 19 (33%) patients changed as follows: the etiological diagnosis of ARF was revised in six cases; of which, five cases with false-negative CMT results were corrected for *P. jirovecii* pneumonia (Nos. 18, 19, 26, 33, and 40), and remaining one case with false-positive CMTs was modified to rheumatic disease activity (No. 49). Additionally, the causative pathogens were modified in 13 patients. Among them, two patients with *A. baumannii* detected by CMTs were finally diagnosed with viral pneumonia (Nos. 6 and 20), four patients diagnosed with single infection by CMTs were changed to mixed infection (Nos. 8, 13, 20, and 41), and seven patients with polymicrobial pneumonia were supplemented with additional pathogens (Nos. 11, 12, 31, 34, 35, 47, and 58). Accordingly, the empiric antimicrobial agents were modified in 14 (24%) patients based on mNGS results, including rescue treatment in 8 patients (Nos. 6, 8, 11, 18, 26, 33, 34, and 47) (mainly for *P. jirovecii*, *Rhizopus* spp., and viruses) and de-escalation or removal in 11 patients (Nos. 6, 18, 20, 26, 33, 34, 40, 49, 50, 55, and 58), and, finally, 4 patients improved (Nos. 8, 33, 49, and 55). In particular, a case of false-positive CMT result was finally diagnosed as dermatomyositis-related ILD and received “pulse” methylprednisone combined with immunoglobulin and tocilizumab, with improved prognosis (No. 49) (**Supplementary Table S2**).

## Discussion

Accurate and rapid etiological diagnosis is urgently needed due to the high mortality rate of rheumatic patients with ARF. The effectiveness of mNGS in detecting respiratory pathogens in this population remains largely unexplored. Early studies only reported its ability to bacteria and virus identification (Parize et al., 2017; Langelier et al., 2018). In recent years, clinicians have been increasingly aware of its advantage for opportunistic pathogens (Pan et al., 2019; Zhang et al., 2019). However, small-scale studies demands further investigation.

This study, to our knowledge, was the largest to evaluate the utility of mNGS in a cohort of critically rheumatic patients with suspected pneumonia. By comparing mNGS and CMTs in a pairwise manner, we found that mNGS not only had high NPV in differentiating pneumonia from non-infectious etiologies but also had excellent accuracy in pathogen identification, which may contribute to clinical decision-making. We also demonstrated that its superiority over different types of pathogens depended on the comprehensiveness of CMTs and doctors’ foreknowledge of potential pathogens, which may facilitate a suitable application scenario for clinicians.

Determining the etiology of ARF in rheumatic patients was extremely challenging. According to our data, mNGS exhibited sufficient sensitivity to exclude infection when the result is negative. This was consistent with previous studies on ICPs, which revealed an NPV of more than 98% (Parize et al., 2017; Langelier et al., 2018). This advantage was particularly important for rheumatic patients, as their lung involvement may be caused by a variety of infectious or non-infectious etiologies, and mistaken diagnosis can lead to serious clinical consequences (Janssen et al., 2002; Falagas et al., 2007; Papiris et al., 2016). mNGS may be used as a “rule-out” assay to exclude infection (Naccache et al., 2015) and provide clues for rapid etiological diagnosis. Just like our patient No. 49, timely negative mNGS result successfully prompted the specific treatment of rheumatic disease-related lung involvement.

Another distinct superiority of mNGS over CMTs was an improvement in pathogen detection. Although this has been reported by previous studies on ICPs (Parize et al., 2017; Langelier et al., 2018; Pan et al., 2019; Li et al., 2020), unlike them, we not only revealed a wide range of pathogens and complex patterns of infections but also evaluated the concordance with the gold standard and the possible reasons for discrepant results, which were often absent in the above studies. We found that mNGS had better sensitivity to various pathogens identification than CMTs, especially opportunistic pathogens, while the false-positive rate was similar. In addition, mNGS had excellent concordance with the gold standard in the multiple pathogens identification for individual patients. On the contrary, CMTs were more likely to omit pathogens and underdiagnose co-pathogens, which may be related to its fundamental limitations, that is, it required multiple microbial tests (such as culture, staining, antigen, and PCR) and doctors’ foreknowledge of possible pathogens (Di Franco et al., 2017; Langelier et al., 2018; Azoulay et al.,2020). In the present study, the possible reasons for false-negative CMTs were as follows: 1) clinicians did not order relevant PCR tests or items for some viruses were not carried out (mainly as HHV and coronavirus); 2) patients were exposed to influenza treatment before sample collection; (3) specimen has low pathogen loads or improper extraction technology (e.g., *P. jirovecii* and *Rhizopus* spp.). It can be seen that the advantages of mNGS in a single assay were very beneficial to rheumatic patients who were more prone to complicated opportunistic or polymicrobial infection.

In terms of pathogen types, we found that mNGS was superior to CMTs in viral detection, which was consistent with previous reports (Di Franco et al., 2017; Langelier et al., 2018; Pan et al., 2019), and the poor accuracy of CMTs was mainly because physicians did not order relevant PCR tests for all viruses on every patient or the items of some viruses were not routinely carried out in our hospital. This also reflected the real clinical situation, that is, due to the wide variety of viruses, it was difficult to check them one by one, and not all viruses had routine identification items, especially rare ones. Notably, there was no agreement on the advantages of mNGS for fungi detection. Some studies revealed that mNGS had an overall superior detection rate to culture (Pan et al., 2019; Huang et al., 2021), while others noted the comparable detection rate between mNGS and conventional tests (OR, 1.42; P = 0.46) (Fang et al., 2020). This divergence may be attributable to different specimen sources, different diseases, and different diagnostic tests. For example, some studies did not carry out the items of antigen detection, PCR, or even GMS (Pan et al., 2019; Huang C et al., 2021), but the examination package that we used can comprehensively evaluate the diagnostic performance of different methods. Our results demonstrated that mNGS and CMTs were comparable in overall fungal detection and slight differences in identifying specific fungal strains. mNGS can significantly improve the poor sensitivity of GMS to *P. jirovecii*, which was also higher than PCR, although the difference was not significant due to the small number of patients with *P. jirovecii* pneumonia (*n* = 24). Moreover, in terms of *Aspergillus* spp., mNGS increased the sensitivity rate by approximately 15% in comparison with that of culture but was inferior to the combination of GM test and culture. This may be related to the difficulty of DNA extraction from the thick polysaccharide cell wall (Bittinger et al., 2014; Clarke et al., 2018; Nilsson et al., 2019). In recent years, it has been found that mNGS may not be the best method to detect *Aspergillus* spp., and detection of cell wall components, such as GM antigen, has an important auxiliary diagnostic value (Hoenigl et al., 2014; Patterson et al., 2016). Overall, we believed that mNGS may be more valuable when some conventional tests are not available (such as GM test and PCR for *P. jirovecii* and viruses) or physicians lack consideration of potential pathogens, which may help to determine the appropriate clinical application scenarios due to current high cost.

We also preliminarily explored the potential benefits of mNGS in diagnosis and treatment. It should be noted that its impact on clinical decision-making (de-escalated or escalated) depended on the empirical antibiotic strategy adopted by each hospital. For instance, we adopted a combined antibiotic regimen covering the most common pathogens (such as bacteria, CMV, and *P. jirovecii*), the treatment changes were less than those reported in other studies, which reported that more than half of patients changed their treatment according to the mNGS result (Miao et al., 2018; Huang et al., 2021). However, it should not be ignored that a considerable percentage of our patients were still suspected of inappropriate empirical antibiotic usage, including 40% of patients (*n* = 23) whose pathogens were uncovered by empirical antibiotic regimens (mainly for molds and viruses) and 86% of patients (*n* = 50) who overused antibiotics (results not shown, see **Supplementary Table S2**). Given that combined treatment has potential side effects (e.g., drug toxicity and resistance), the high accuracy and faster turnaround time of mNGS may help clinicians formulate a targeted therapeutic schedule to avoid antibiotic overuse.

Our study has several limitations. First, single-center retrospective study with a small sample size demands further investigation with larger cohorts. Second, none of our patients was diagnosed by lung biopsy, which may lead to overdiagnosis. In particular, the utility of CMV-DNA viral load measurement have not been standardized. Considering that our patients had severe impairment of cell-mediated immunity (median CD4^+^ T cell counts of 99 × 10^6^/L, **Table 1**) and the median viral load (2.52 × 10^5^ copies/ml, **Table 2**) was similar to or higher than that reported in previous studies (Xue et al., 2016; Lee et al., 2017) indicated a greater likelihood of infection. But not negatively, it was very difficult to distinguish between latent and active CMV infection, and many clinical manifestations of CMV pneumonia may be similar to other infections, so it is necessary to further study the significance of CMV DNA. Third, to date, no uniform standards have been reported regarding the interpretation of mNGS results. We interpreted with caution in combination with clinical composite diagnosis and orthogonal confirmation. However, misjudgment cannot be completely avoided. Last, prospective studies are needed to further evaluate its medical benefits (i.e., clinical decision-making, antibiotic consumption, cost-effect, and outcome) in the management of critically rheumatic patients.

In summary, the excellent breadth and accuracy of mNGS in pathogen identification make it to be a promising diagnostic technique in critically rheumatic patients with suspected pneumonia. It also has potential significance for tailoring antimicrobial regimens. In the current clinical practice, mNGS may need to be combined with a fungal antigen test due to its low sensitivity to *Aspergillus* spp.

## Data availability statement

The datasets presented in this study can be found in online repositories. The names of the repository/repositories and accession number(s) can be found in the article/**Supplementary Material**.

## Ethics statement

The studies involving human participants were reviewed and approved by the ethics committee of Peking Union Medical College Hospital. The ethics committee waived the requirement of written informed consent for participation.

## Author contributions

YS and BD conceived and designed this study. Material preparation and data collection by YS, J-MP and H-YQ. YS, J-MP, H-YQ and BD wrote the manuscript. All authors contributed to the article and approved the submitted version.

## Funding

This study was supported by the Special Fund for Clinical Research of Wu Jieping Medical Foundation (Grant No. 320.6750.18428] and Clinical fund of Peking Union Medical College Hospital (Grant No. ZC201904394).

## Acknowledgments

We thank the patients for cooperating with our investigation. We also thank the staff of BGI for providing detailed sequencing data and operation process.

## Conflict of interest

The authors declare that the research was conducted in the absence of any commercial or financial relationships that could be construed as a potential conflict of interest.

## Publisher’s note

All claims expressed in this article are solely those of the authors and do not necessarily represent those of their affiliated organizations, or those of the publisher, the editors and the reviewers. Any product that may be evaluated in this article, or claim that may be made by its manufacturer, is not guaranteed or endorsed by the publisher.
