# Supplementary material for: Metagenomic next-generation sequencing: A promising tool for diagnosis and treatment of suspected pneumonia in rheumatic patients with acute respiratory failure: Retrospective cohort study
Source: Front Cell Infect Microbiol. 2022 Aug 3;12:941930. doi: 10.3389/fcimb.2022.941930 (PMC9381725; doi:10.3389/fcimb.2022.941930)
Supplement: Supplementary file 1 [file Table_1.docx]

**Supplementary Table S1.** Interpretation of the results of combined microbiological tests

| Pathogen | Definition of clinically–relevant microorganisms |
| --- | --- |
| Bacteria | > 1 positive moderate to heavy growths of bacteria from BALF/TA (leukocytes > 25 per high-power microscopic field and few epithelial cells < 10 per highpower microscopic field); oral commensal organisms are considered as contaminants. |
| Atypical pathogens | Positive PCR for *Legionella*, *Mycoplasma*, *Chlamydia* from BALF; or |
|  | Elevated serum levels of IgM against *C. pneumoniae* (≥ 1:64) or  *M. pneumoniae* (any positive titre) or a four fold increase in IgG titres; or |
|  | Positive urinary antigen for *L. pneumophila* |
| *Mycobacterium* spp. | Positive anti-fast staining from BALF/TA; or Positive PCR test from BALF; or  Positive culture from BALF/TA |
| Fungi |  |
| *Aspergillus* spp. | Positive culture from BALF/TA or single serum GM test ≥1.0 or BALF ≥1.0 or single serum GM test ≥0.7 and BALF ≥0.8 combined with host factors and imaging findings (as criteria for probable pulmonary aspergillosis) |
|  |  |
| Fungi other than  *Aspergillus* spp. | Positive culture for fungi other than *Aspergillus* from BALF/TA combined with host factor and clinical feature; and except for colonization; *Candida* spp. are disregarded unless they are deemed significant by the managing physician |
| *P. jirovecii* | Detection of cyst of *Pneumocystis* by Gomori's  methenamine silver stain (as proven criteria); or |
|  | Positive PCR of *P. jirovecii* from BALF combined with respiratory symptoms (such as cough, dyspnea, and hypoxemia) and classic/compatible radiological findings (as criteria for probable *P. jirovecii* pneumonia) |
| *Cryptococcus* | Positive culture from BALF/TA; or visualization of capsule by India ink stain in BALF/TA |
|  |  |
| Viruses | Positive PCR for viruses of interest combined with clinical and radiology features; or positive antigen for *influenza* A/B |

*BALF: bronchoalveolar lavage fluid; GM: galactomannan; PCR: polymerase-chain-reaction; TA: tracheal aspirate*
