# Supplementary material for: Metagenomic next-generation sequencing: A promising tool for diagnosis and treatment of suspected pneumonia in rheumatic patients with acute respiratory failure: Retrospective cohort study
Source: Front Cell Infect Microbiol. 2022 Aug 3;12:941930. doi: 10.3389/fcimb.2022.941930 (PMC9381725; doi:10.3389/fcimb.2022.941930)
Supplement: Supplementary file 2 [file Table_2.docx]

**Supplementary Table S2.**  Detailed information of 58 critically rheumatic patients with suspected pneumonia

| Patient No. | Type of disease | CMTs results | mNGS results | SMRN | Coverage (%) | | Relative abundance (%) | Clinical composite diagnosis | Empirical antibiotics | Treatment changes  based on mNGS results alone | Outcome |
| --- | --- | --- | --- | --- | --- | --- | --- | --- | --- | --- | --- |
| 1 | RA | Serum-GM 1.16 ;  BALF-GM 1.28;  BALF-qPCR for CMV 740,000 copies/ml;  BALF-PCR for Pj (+) | CMV | 270 | 8.6 | | 83.57 | *Aspergillus,*  CMV, Pj | Ceftriaxone, TMP-SMZ, Moxifloxacin and Foscarnet | - | Death |
| 2 | DM | Culture *P. aeruginosa* | *P. aeruginosa* | 10,469 | 17.6 | | 95.13 | *P. aeruginosa,* | Piperacillin/tazobactam | *-* | Death |
|  |  | BALF-PCR for Pj (+) | Pj | 7 | 0.0998 | | 42.18 | Pj, *Aspergillus* | Moxifloxacin and |  |  |
|  |  | Serum-GM 3.85  BALF-GM 3.95 | *Aspergillus* | 768 | 0.4261 | | 74.39 |  | TMP-SMZ |  |  |
| 3 | AASV | BALF culture *P. aeruginosa*  BALF- PCR for Pj (+)  BALF qPCR for CMV 35,000 copies/ml | *P. aeruginosa*  Pj  CMV | 3626  48  38 | 17.24  0.0783  23.28 | | 53.23  63.45  42.89 | *P. aeruginosa* | Moxifloxacin, | - | Alive |
|  |  |  |  |  |  |  |  | Pj; CMV | Ceftriaxone, Ganciclovir |  |  |
|  |  |  |  |  |  |  |  |  | Prophylactic TMP-SMZ |  |  |
| 4 | Anti-Jo-1  syndrome | Smear Gram-positive cocii  BALF culture *Aspergillus,*  Serum-GM 0.75, BALF-GM>5;  BALF Influenza B PCR(+) | *E. faecium*  *Aspergillus*  *Influenza* B | 6100  355  92 | 11.3  1.74  50.96 | | 44.79  78.22  97.09 | *E. faecium,*  *Aspergillus*,  *Influenza* B | Imipenem  Foscarnet,TMP-SMZ,  Oseltamivir | - | Death |
|  |  |  |  |  |  |  |  |  |  |  |  |
|  |  |  |  |  |  |  |  |  |  |  |  |
| 5 | Sjögren's  syndrome | Anti-fast staining (+); Culture and PCR for *M. tuberculosis*(+); Serum-GM 0.87; BALF GM 2.72 | MTBC | 6 | 0.21 | | 31.3 | *M. tuberculosis*;  *Aspergillus.* | Moxifloxacin and Voriconazole | *-* | Alive |
| 6 | IPAF | BALF culture *A.* *baumannii* ^†^ | *Parainfluenza*  *A. baumannii* ^‡^ | 474  674 | 70.2  1.35 | | 98.67  35.13 | *Parainfluenza* | Cefoperazone/Sulbactam, Moxifloxacin | Add Oseltamivir Discontinued other antibiotics | Alive |
|  |  |  |  |  |  |  |  |  |  |  |  |
| 7 | DM | Serum GM 0.72;  BALF GM 1.02;  BALF- PCR for *Pj* (+) | Pj | 447 | 0.3292 | | 96.38 | *Aspergillus*, Pj | TMP-SMZ, Oseltamivir, Moxifloxacin and Ceftriaxone | - | Alive |
| 8 | RA | BALF qPCR for CMV 41,000 copies/ml | *Influenza* B  CMV | 103  89 | 80.9  2.5 | | 90.4  37.7 | *Influenza B;*  CMV | TMP-SMZ, Ganciclovir,  Cefoperazone/Sulbactam and Moxifloxacin, Prophylactic Oseltamivir | Added Oseltamivir; | Alive |
|  |  |  |  |  |  |  |  |  |  |  |  |
| 9 | DM | Culture *A. baumannii and* | *A. baumannii ;*  *K. pneumoniae*  Pj | 3858  1687  16 | 18.25  28.71  0.0113 | | 55.11  38.94  68.04 | *A. baumannii*  *K. pneumoniae*  Pj*; Aspergillus* | TMP-SMZ and | - | Death |
|  |  | *K.pneumoniae;*  Serum GM 3.5; |  |  |  |  |  |  | Cefoperazone/Sulbactam |  |  |
|  |  | BALF- PCR for Pj (+) |  |  |  |  |  |  |  |  |  |
| 10 | AOSD | BALF- PCR for Pj (+) | Pj*,* | 2726  46 | 1.67  0.9916 | | 99.31  49.02 | Pj*; CMV* | TMP-SMZ, Moxifloxacin | - | Alive |
|  |  | BALF qPCR for CMV 200,000 copies/ml | CMV |  |  |  |  |  | and Foscarnet, Oseltamivir |  |  |
| 11 | SLE | culture *A. baumannii;* | *A. baumannii* | 8185 | 14.03 | | 87.73 | *A. baumannii ;* | Cefoperazone/Sulbactam Moxifloxacin, Ganciclovir | Added Amphotericin B; | Death |
|  |  | BALF culture *Aspergillus* | *Aspergillus* | 2213 | 0.4065 | | 53.15 | *Aspergillus*; |  |  |  |
|  |  |  | *Rhizopus*spp | 647 | 0.2132 | | 39.54 | *Rhizopus spp*. |  |  |  |
| 12 | AASV | BALF *culture C.neoformans*  BALF PCR for *Pj* (+)  BALF qPCR for *CMV* 41,000 copies/ml | *C. neoformans*  Pj  CMV *Coronavirus OC43* | 1749  538  38  17 | 1.6  0.3315  0.2335  2.62 | | 87.95  99.24  51.83  77.75 | *C.neoformans;*  Pj; CMV  *Coronavirus* | Ceftriaxone, Moxifloxacin  TMP-SMZ and Ganciclovir | *-* | Death |
|  |  |  |  |  |  |  |  |  |  |  |  |
|  |  |  |  |  |  |  |  |  |  |  |  |
| 13 | SLE | BALF- PCR for Pj (+) | HHV type-1 | 11 | 0.82 | | 100 | Pj, HHV | Ceftriaxone, Ganciclovir, Moxifloxacin, TMP-SMZ | - | Death |
| 14 | SLE | BALF- PCR for Pj (+) | Pj | 16 | 0.0046 | | 87.67 | Pj | Ceftriaxone, Ganciclovir, Moxifloxacin, TMP-SMZ | - | Alive |
| 15 | IPAF | Serum *M. Pneumoniae*  Ig M ≥1:160 | *M. Pneumoniae* | 51 | 73.17 | | 86.18 | *M. Pneumoniae* | Moxifloxacin, Ceftriaxone | - | Alive |
| 16 | AASV | Serum GM 1.99  BALF culture *Aspergillus*  BALF qPCR for CMV 455,000 copies/ml | *Aspergillus*  CMV  Pj | 153 | 1.6 | | 87.95 | *Aspergillus;* Pj; | TMP-SMZ and | - | Death |
|  |  |  |  | 791  2506 | 70.2  0.908 | | 93.67  89.5 | CMV | Moxifloxacin, Ceftriaxone |  |  |
| 17 | SLE | Negative | Negative |  |  | |  | SLE related ILD | TMP-SMZ, Cefoperazone/Sulbactam | - | Alive |
| 18 | Sjögren's  syndrome | Negative | Pj | 1060 | 1.29 | | 99.82 | Pj | Ceftriaxone, Moxifloxacin  prophylactic TMP/SMZ | Added TMP-SMZ  Discontinued other antibiotics | Death |
| 19 | SLE | Negative | Pj | 448 | 0.3429 | | 97.71 | Pj | Ceftriaxone, Moxifloxacin and TMP-SMZ | Removed Ceftriaxone Moxifloxacin | Death |
| 20 | Systemic vasculitis | BALF culture *A. baumannii*^†^ | CMV | 189 | 1.97 | | 34.25 | CMV; HHV | Cefoperazone/sulbactam,  Prophylactic TMP-SMZ  and Ganciclovir | Removed Cefoperazone/  sulbactam | Death |
|  |  |  | HHV type-1 | 18 | 59.25 | | 33.19 |  |  |  |  |
|  |  |  | HHV type-6 | 38 | 54.57 | | 32.38 |  |  |  |  |
|  |  |  | *A. baumannii*^‡^ | 889 | 11.23 | | 33.57 |  |  |  |  |
| 21 | UCTD | BALF qPCR for CMV 160,000 copies/ml; | CMV  Pj | 28  6876 | 0.1853  0.0607 | | 58.47  96.3 | CMV, Pj | TMP-SMZ, Moxifloxacin, | - | Death |
|  |  | BALF- PCR for Pj (+) |  |  |  |  |  |  | Ceftriaxone, Ganciclovir |  |  |
| 22 | Systemic vasculitis | BALF culture *A. baumannii,*  *P. aeruginosa and* A*spergillus spp.*; | *A. baumannii ,* | 26,832 | 4.17 | | 45.79 | *A. baumanii,*  *P. aeruginosa,*  *Aspergillus;*  CMV | Impenem, TMP-SMZ,  Ganciclovir,  Oseltamivir | - | Death |
|  |  |  | *P. aeruginosa,*  *Aspergillus*  CMV | 51,947  9  19 | 6.63  0.0788  1.62 | | 47.14  37.81  79.31 |  |  |  |  |
|  |  | Serum GM 1.99, BALF GM >5 |  |  |  |  |  |  |  |  |  |
|  |  | BALF qPCR for CMV 107,000 copies/ml |  |  |  |  |  |  |  |  |  |
| 23 | SLE | BALF culture *A. baumannii* ^†^ | *A. baumannii* ^‡^ | 1190 | 2.37 | | 33.7 | SLE related ILD | Cefoperazone-Sulbactam,  Prophylactic TMP-SMZ | - | Alive |
| 24 | Systemic vasculitis | BALF and blood culture *Salmonella;* BALF qPCR for CMV 518,000 copies/ml | *Salmonella*  CMV | 2997  155 | 85.52  1.37 | | 73.19  95.04 | *Salmonella;*  CMV | Cefoperazone-Sulbactam,  Prophylactic TMP-SMZ | - | Alive |
|  |  |  |  |  |  |  |  |  |  |  |  |
| 25 | SLE | Negative | Negative |  |  | |  | SLE activity related DAH | Prophylactic TMP-SMZ, Moxifloxacin, Ceftriaxone |  | Alive |
| 26 | SLE | Negative | Pj | 19 | 0.0137 | | 82.52 | Pj | Ceftriaxone, Moxifloxacin Ganciclovi and prophylactic TMP-SMZ | Added TMP-SMZ;  Removed other antibiotics | Death |
| 27 | SLE | BALF- PCR for Pj (+); | Pj; | 6065 | 1.29 | | 98.22 | Pj, CMV | Ceftriaxone, Ganciclovi | - | Death |
|  |  | BALF qPCR for CMV 530,000 copies/ml | CMV | 277 | 1.97 | | 96.04 |  | Moxifloxacin, TMP-SMZ |  |  |
| 28 | DM | Negative | Negative |  |  | |  | DM related ILD | TMP-SMZ, Ceftriaxone |  | Death |
| 29 | AASV | BALF culture *Aspergillus*;  BALF qPCR for CMV 7,600,000 copies/ml | *Aspergillus*  CMV  *S. epidermidis ^‡^* | 7  1013  881 | 0.0041  48.6  2.03 | | 70.17  91.56  31.37 | *Aspergillus;* | Ceftriaxone, Moxifloxacin | - | Death |
|  |  |  |  |  |  |  |  | CMV | TMP-SMZ, Ganciclovi |  |  |
| 30 | DM | BALF- PCR for Pj (±);  Culture *A. baumannii* ^†^ | Pj  *A. baumannii*^‡^ | 79  783  19,579  12,895  208  915  33  17 | 0.0041  3.17  13.93  7.89  1.91  0.5722  0.4852  0.3714 | | 70.17  30.51  57.25  37.59  38.22  99.52  43.74  35.7 | Pj | Ceftriaxone, Moxifloxacin | - | Death |
|  |  |  |  |  |  |  |  |  | TMP-SMZ |  |  |
| 31 | AASV | BALF culture *K. pneumoniae* | *K. pneumoniae*  *S. pneumoniae*  *Aspergillus*  Pj  CMV  HHV type-1 |  |  |  |  | *K.pneumoniae*  *S. pneumonia;*  *Aspergillus*;  Pj;CMV;HHV-1 | Imipenem, TMP-SMZ  Ganciclovir | - | Death |
|  |  | BALF culture A*spergillus;* |  |  |  |  |  |  |  |  |  |
|  |  | Serum GM 2.9; BALF-GM 3.3 |  |  |  |  |  |  |  |  |  |
|  |  | BALF- PCR for Pj (+) |  |  |  |  |  |  |  |  |  |
|  |  | BALF qPCR for CMV 24,000 |  |  |  |  |  |  |  |  |  |
| 32 | SLE | Negative | Negative |  |  | |  | SLE activity related DAH | Cefoperazone-Sulbactam,TMP-SMZ and Ganciclovir |  | Alive |
| 33 | RA | Negative | *Pj* | 7 | 0.0096 | | 90.45 | *Pj* | Ceftriaxone and Moxifloxacin | Added TMP-SMZ, Removed other antibiotics | Alive |
| 34 | SLE | Gomori's methenamine silver stain (+); | Pj  CMV  B type-RSV  *Influenza B* | 9585  49  467  39 | 1.6  0.9716  1.96  0.3197 | | 97.95  30.27  32.75  33.09 | Pj; CMV;  B type-RSV;  *Influenza* B | Ceftriaxone, moxifloxacin, Ganciclovir, TMP-SMZ | Added therapeutic Oseltamivir; Removed Ceftriaxone, moxifloxacin | Death |
|  |  | BALF- PCR for Pj (+)；  BALF qPCR for CMV 303,000 copies/ml |  |  |  |  |  |  |  |  |  |
|  |  |  |  |  |  |  |  |  | Prophylactic Oseltamivir |  |  |
| 35 | RA | BALF PCR for influenza A (+); BALF-GM>5; | *Influenza A*  *Aspergillus*  *A.baumannii*  *Coronavirus 229E* | 132  52  14,849  17 | 7.54  0.0129  19.57  0.7321 | | 47.37  74.4  70.05  44.8 | *influenza A;*  *Aspergillus;*  *A.baumannii ;*  *Coronavirus* | Cefoperazone-Sulbactam,TMP-SMZ, Ganciclovir, | - | Death |
|  |  | BALF culture *Aspergillus and* *A. baumannii* |  |  |  |  |  |  |  |  |  |
|  |  |  |  |  |  |  |  |  |  |  |  |
|  |  |  |  |  |  |  |  |  |  |  |  |
| 36 | DM | BALF- PCR for Pj (+) | Pj | 1506 | 1.3141 | | 99.5 | Pj | Ceftriaxone,TMP/SMZ and moxifloxacin | - | Death |
| 37 | SSc | Negative | Negative |  |  | |  | SSc related ILD | TMP-SMZ, Ceftriaxone and Moxifloxacin | - | Death |
| 38 | AASV | BALF- PCR for Pj (±) | Pj  *A. baumannii*^‡^  *P. aeruginosa* ^‡^ | 18  778  669 | 0.3292  7.18  5.39 | | 96.17  33.75  31.17 | Pj | TMP-SMZ, Ceftriaxone and Moxifloxacin | - | Death |
|  |  | Culture A. *baumannii* ^†^ |  |  |  |  |  |  |  |  |  |
| 39 | DM | Negative | Negative |  |  | |  | RP-ILD | TMP-SMZ, Ceftriaxone, Moxifloxacin |  | Death |
| 40 | SLE | Negative | Pj | 7 | 0.0038 | | 90.31 | Pj | TMP-SMZ, Ceftriaxone, Moxifloxacin and Ganciclovir | Discontinued other antibiotics other than TMP-SMZ | Death |
| 41 | MCTD | BALF qPCR for CMV107,000 copies/ml | *CMV*  *H. influenzae* | 11  3837 | 0.9916  4.78 | | 37.02  58.79 | CMV  *H. influenzae* | TMP-SMZ, Ceftriaxone and Ganciclovir | - | Alive |
|  |  |  |  |  |  |  |  |  |  |  |  |
| 42 | DM | Negative | Negative |  |  | |  | DM related ILD | Moxifloxacin, TMP-SMZ, Ceftriaxone, Ganciclovir | - | Death |
| 43 | SLE | Negative | Negative |  |  | |  | SLE activity related ACPE | Prophylactic TMP-SMZ and Ceftriaxone | - | Alive |
| 44 | AOSD | BALF culture *P. aeruginosa* | *P. aeruginosa* | 5574 | 8.7 | | 85.37 | *P. aeruginosa* | Meropenem and prophylactic TMP-SMZ | - | Alive |
| 45 | RA | BALF culture *A. baumannii* | *A. baumannii* | 10,899 | 39.15 | | 70.53 | A. *baumannii* | Cefoperazone-Sulbactam, Prophylactic TMP-SMZ | - | Alive |
| 46 | SLE | Negative | Negative |  |  | |  | SLE activity related DAH | Cefoperazone-Sulbactam  Ganciclovir and Prophylactic TMP-SMZ | - | Alive |
| 47 | SLE | BALF culture *A.* *baumannii* | *A. baumannii;* | 1,173,458 | | 13.8 | 58.19 | *A. baumannii ;*  *Aspergillus*  *Rhizopus spp;*  HHV | Cefoperazone-Sulbactam | Added Amphotericin B | Death |
|  |  | Serum GM 1.81,  BALF GM 3.91 | *E. aerogenes* ^‡^  *Aspergillus*  *Rhizopus* spp.  HHV type-1 | 887  28  13  87,437 | 1.78  0.0465  0.0232  90.9 | | 31.47  53.15  37.65  84.77 |  | Ganciclovir, TMP-SMZ |  |  |
|  |  |  |  |  |  |  |  |  |  |  |  |
|  |  |  |  |  |  |  |  |  |  |  |  |
| 48 | SLE | Negative | Negative |  |  | |  | SLE activity related DAH | Cefoperazone-SulbactamProphylactic TMP-SMZ | *-* | Alive |
| 49 | DM | BALF culture A*spergillus spp* ^†^ | Negative |  |  | |  | DM related RP-ILD | TMP-SMZ, Moxifloxacin and Voriconazole | Immunoglobulin, pulse methylpred-nisone and Tocilizumab; removed antibiotics | Alive |
| 50 | SLE | BALF-GM 2.4  BALF- PCR for Pj (±) ^†^ | A*spergillus* | 11 | 0.079 | | 74.48 | *Aspergillus* | TMP-SMZ, Moxifloxacin | Removed TMP-SMZ and Moxifloxacin | Death |
| 51 | SLE | Negative | Negative |  |  | |  | ACPE | Cefoperazone-Sulbactam,  prophylactic TMP-SMZ | - | Alive |
| 52 | DM | Negative | Negative |  |  | |  | DM related ILD | TMP-SMZ, Ceftriaxone, Moxifloxacin and Ganciclovir | *-* | Death |
| 53 | DM | BALF qPCR for CMV 740,000 copies/ml | CMV | 28,159 | 58.96 | | 98.17 | CMV | TMP-SMZ, Ceftriaxone, Moxifloxacin, Ganciclovir | - | Death |
| 54 | IPAF | BALF culture *P. aeruginosa* | *P. aeruginosa* | 18,444 | 15.3 | | 84.79 | *P. aeruginosa* | Cefoperazone-Sulbactam and Moxifloxacin | - | Death |
| 55 | AASV | PCR (+) for *M. tuberculosis*;  Culture *M. tuberculosis and Aspergillus spp*;  Serum-GM 0.7;  BALF qPCR for CMV 1070^†^ | MTBC | 9 | 0.020 | | 41.88 | *M. tuberculosis*;  *Aspergillus*, | Meropenem, Ganciclovir, prophylactic TMP-SMZ | Discontinued Ganciclovir and Meropenem | Alive |
| 56 | Systemic vasculitis | BALF qPCR for CMV 500,000 copies/ml; | CMV  Pj | 147  529 | 3.13  0.3147 | | 67.36  98.5 | CMV;  Pj | TMP-SMZ, Ganciclovir Moxifloxacin, Ceftriaxone | - | Death |
|  |  | BALF- PCR for Pj (+) |  |  |  |  |  |  |  |  |  |
| 57 | SLE | Negative | Negative |  |  | |  | SLE activity related DAH | Prophylactic TMP-SMZ, Cefoperazone-Sulbactam |  | Death |
| 58 | AOSD | BALF PCR for Pj (+)  BALF qPCR for CMV 1400 copies/ml ^†^ | Pj  HHV type-1 | 2726  92 | 1.67  50.37 | | 99.31  97.09 | Pj, HHV | TMP-SMZ, Moxifloxacin, Ceftriaxone, Ganciclovir | Discontinued Ganciclovir, Moxifloxacin, Ceftriaxone | Alive |

AASV: ANCA associated systemtc vasculitis; ACPE: acute cardiogenic pulmonary edema; AOSD: Adult onset Still’s disease; BALF, bronchoalveolar lavage fluid; CMV, cytomegalovirus; CMTs: combined microbiological tests; DAH: diffuse alveolar hemorrhage; DM: dermatomyositis; GM: galactomannan; HHV: *human herpes virus*; ILD: interstitial lung disease; IPAF: interstitial pneumonia with autoimmune features; MCTD: mixed connective tissue disease; mNGS: metagenomic next-generation sequencing; MTBC: *Mycobacterium tuberculosis* complex; Pj: *Pneumocystis jirovecii*; PCR: polymerase chain reaction; qPCR: Quantitative Real-time PCR; RA: rheumatoid arthritis; RP-ILD: rapid progressive interstitial lung disease; RSV: *respiratory syncytial virus*; SLE: systemic lupus erythematosus; SSc: systemic sclerosis; TMP-SMZ: trimethoprim-sulfamethoxazole; UCTD: undifferentiated connective tissue disease.

^†^: Clinicians concluded that the CMTs results were false positive and the possible reasons included colonization (5 cases of *A. baumannii* in No. 6, 20, 23, 30 and 38, and one of *P. jirovecii* in No.50), possible contamination (one of *Aspergillus* in No. 49) and likelihood of latent infection (2 of CMV in No. 55 and 58)

^‡^ : Bacteria detected by mNGS were considered as colonization, including *A. baumannii* in patients No. 6, 20, 23 and 30; *A. baumannii and P. aeruginosa* in patient No.38,  *S. epidermidis* in patient No.29, and *E. aerogenes* in patient No. 47.

**Table S3**. Diagnostic performance of combined microbiological tests and metagenomic next-generation sequencing at the pathogen type level

|  | mNGS | | | | | CMTs | | | | |
| --- | --- | --- | --- | --- | --- | --- | --- | --- | --- | --- |
|  | Sensitivity | Specificity | PPV | NPV | Accuracy | Sensitivity | Specificity | PPV | NPV | Accuracy |
| **Bacteria** | 100  (77.1-100) | 83.3  (66.5-93.0) | 73.9  (51.3-88.9) | 100  (85.9-100) | 88.7  (76.9-94.9) | 94.1  (69.2-99.7) | 86.1  (69.7-94.8) | 76.2  (52.5-90.9) | 96.9  (82.0-99.8) | 88.7  (81.1-95.8) |
| **Fungi** | 87.9  (70.9-96.0) | 100  (88.0-100) | 100 (85.4-100) | 83.3  (61.8-94.5) | 92.5  (82.4-98.7) | 84.8  (67.3-94.3) | 90.0  (66.9-98.2) | 93.3  (76.5-98.8) | 78.3  (55.8-91.7) | 86.8  (71.1-93.2)) |
| *P. jirovecii* | 91.7  (71.5-98.5) | 100  (85.4-100) | 100  (81.5-100) | 93.5  (77.2-98.9) | 96.2  (90.9-99.2) | 79.2  (57.3-92.1) | 96.6  (80.4-99.9) | 95.1  (73.1-99.7) | 84.8  (67.3-94.3) | 88.7  (73.4-96.7) |
| *Aspergillus spp.* | 66.7  (38.7-87.0) | 94.7  (80.9-99.1) | 83.3  (50.8-97.0) | 87.8  (72.9-95.6) | 87.8  (77.4-96.2) | 100  (74.7-100) ^*^ | 97.3  (84.6-99.9) | 93.8  (67.7-99.7) | 100  (88.2-100) | 98.1  (94.3-99.2) |
| **Viruses** | 100  (82.2-100) | 100 (87.7-100) | 100  (82.2-100) | 100  (87.7-100) | 100  (87.9-100) | 56.5  (34.9-76.1) ^*^ | 97.1  (83.4-99.8) | 92.9  (64.2-99.6) | 77.3  (61.8-88.0) ^*^ | 81.0  (62.6-87.7) ^*^ |
| CMV | 100  (77.1-100) | 100  (89.3-100) | 100  (77.0-100) | 100  (89.3-100) | 100  (89.6-100) | 94.1  (69.2-99.7) | 95.1  (82.2-99.2) | 88.9  (63.9-98.1) | 97.5  (85.3-99.9) | 94.8  (83.9-99.7) |
| Others viruses | 100  (69.9-100) | 100  (90.4-100) | 100  (69.9-100) | 100  (90.3-100) | 100  (90.7-100) | 16.7  (3.0-49.1) ^*^ | 100  (90.4-100) | 100  (19.8-100) | 82.1  (69.2-90.7) | 82.8  (68.9-96.7) ^*^ |

Data are presented as % (95% CI)

CI: confidence interval; CMTs: combined microbiological tests; CMV: cytomegalovirus; mNGS: metagenomic next-generation sequencing; NPV: negative predictive value; PPV: positive predictive value

^*^ The difference in the parameter was significant between CMTs and mNGS based on the *McNemar* test (*P* < 0.05).
