# Supplementary material for: Metagenomic next-generation sequencing: A promising tool for diagnosis and treatment of suspected pneumonia in rheumatic patients with acute respiratory failure: Retrospective cohort study
Source: Front Cell Infect Microbiol. 2022 Aug 3;12:941930. doi: 10.3389/fcimb.2022.941930 (PMC9381725; doi:10.3389/fcimb.2022.941930)
Supplement: Supplementary file 3 [file Table_3.docx]

**Appendix 1.** Detailed procedures of bronchoalveolar lavage fluid metagenomic next-generation sequencing

**Sample Acquisition, Processing, and Nucleic Acid Extraction**

Samples of 10 mL bronchoalveolar lavage fluid (BALF) were collected from patients according to standard procedures, and were immediately sent to BGI clinical laboratory Co., Ltd. Initially, a 1.5 ml microcentrifuge tube (containing 0.5 ml BALF sample and 1 g 0.5-mm glass beads) was connected to a horizontal platform on the vortex mixer. Subsequently, the tube was agitated vigorously at 2,800–3,200 rpm for 30 min. Subsequently, the 0.3 ml sample was separated into a new 1.5 ml microcentrifuge tube and DNA/RNA was extracted using the TIA-Namp Micro DNA/RNA Kit (DP316, Tiangen Biotech Beijing, China) according to the manufacturer’s protocol.

**RNA enrichmen**

After mixing 33 microliters of the extracted nucleic acid sample with 7 microliters of the enrichment reaction mixture, incubate on a PCR machine at 37℃ for 10 min, and then perform magnetic bead purification to remove DNA from nucleic acids, thereby improving the concentration of RNA content.

**Reverse transcription and two-strand synthesis**

The unenriched nucleic acid or the enriched nucleic acid is subjected to fragmentation reaction, one-strand synthesis and two-strand synthesis to form double-stranded DNA nucleic acid, then purified by magnetic beads, and the purified DNA is used for DNA library construction.

**Construction of DNA library**

DNA libraries were constructed through an end-repair method in which the adapters were added overnight, and polymerase chain reaction (PCR) amplification was used prior to analysis using an Ion Torrent Proton Sequencer (Life Technologies, Carlsbad, California). The sequencing was performed on the BGISEQ-50/MGISEQ-2000 platform (Fang et al., 2018). The quality of the DNA libraries was assessed using an Agilent 2100 Bioanalyzer (Agilent Technologies, Santa Clara, California) combined with quantitative PCR to measure the adapters before sequencing.

**Sequencing and Bioinformatic Analyses**

High-quality sequencing data were generated by removing low-quality and short-length (< 35 bp) reads, followed by a computational subtraction of human sequences mapped to the human reference genome (hg19) by Burrows–Wheeler alignment. Following completion of the above steps, the remaining sequence data were aligned to the current bacterial, viral, fungal, and protozoan databases, which were downloaded from the National Center Biotechnology Information (ftp://ftp.ncbi.nlm.nih.gov/genomes/). The database used for the present study contained 2,328 bacterial species, 4,189 viral species, 199 fungal species, 135 parasites, and 40 *mycoplasma*/*chlamydia*, which were associated with human diseases.

Reference

Fang C, Zhong H, Lin Y, Chen B, Han M, Ren H, et al. 2018. Assessment of the cPAS-based BGISEQ-500 platform for metagenomic sequencing. *Gigascience* 7, 1-8. doi: 10.1093/gigascience/gix133
